# A Synergic Integration of AIS Data and SAR Imagery to Monitor Fisheries and Detect Suspicious Activities †

**DOI:** 10.3390/s21082756

**Published:** 2021-04-13

**Authors:** Alessandro Galdelli, Adriano Mancini, Carmen Ferrà, Anna Nora Tassetti

**Affiliations:** 1 VRAI Lab, Dipartimento di Ingegneria dell’Informazione, Università Politecnica delle Marche, 60131 Ancona, Italy; a.mancini@univpm.it; 2 CNR-IRBIM, Institute for Marine Biological Resources and Biotechnology, National Research Council, 60125 Ancona, Italy; carmen.ferravega@cnr.it (C.F.); annanora.tassetti@cnr.it (A.N.T.)

**Keywords:** Automatic Identification System, Synthetic Aperture Radar, data integration, machine learning, maritime surveillance

## Abstract

Maritime traffic and fishing activities have accelerated considerably over the last decade, with a consequent impact on the environment and marine resources. Meanwhile, a growing number of ship-reporting technologies and remote-sensing systems are generating an overwhelming amount of spatio-temporal and geographically distributed data related to large-scale vessels and their movements. Individual technologies have distinct limitations but, when combined, can provide a better view of what is happening at sea, lead to effectively monitor fishing activities, and help tackle the investigations of suspicious behaviors in close proximity of managed areas. The paper integrates non-cooperative Synthetic Aperture Radar (SAR) Sentinel-1 images and cooperative Automatic Identification System (AIS) data, by proposing two types of associations: (i) point-to-point and (ii) point-to-line. They allow the fusion of ship positions and highlight “suspicious” AIS data gaps in close proximity of managed areas that can be further investigated only once the vessel—and the gear it adopts—is known. This is addressed by a machine-learning approach based on the Fast Fourier Transform that classifies single sea trips. The approach is tested on a case study in the central Adriatic Sea, automatically reporting AIS-SAR associations and seeking ships that are not broadcasting their positions (intentionally or not). Results allow the discrimination of collaborative and non-collaborative ships, playing a key role in detecting potential suspect behaviors especially in close proximity of managed areas.

## 1. Introduction

In previous centuries, many naturalists believed that fishery resources were inexhaustible and independent of human harvesting, due to their consistency, dispersal capacity and reproductive potential [1,2]. However, the continued exploitation and overfishing observed in many areas of the world have drastically reduced the abundance of fish resources and the Food and Agriculture Organization of the United Nations (FAO) has highlighted the need to manage marine resources so that the rate of harvest is commensurate with their capacity to self-renew [3,4].

For this reason, improving Maritime Domain Awareness (MDA) and sustainable use of oceans, seas and marine resources has grown in importance [5] and requires monitoring tools that can provide observations on the state of fish stocks [6,7,8] and on fishing fleets’ activity [9,10,11].

Presently, fishing activities can be monitored by several systems that can be broadly classified as cooperative and non-cooperative. Cooperative systems rely on self-reporting information from vessels providing details on identification, position and speed; this category includes data from Automatic Identification System (AIS), Long Range Identification and Tracking (LRIT) and the Vessel Monitoring System (VMS). On the other hand, non-cooperative systems employ radar and optical sensors (coastal, shipborne, airborne, and spaceborne) to detect ships from the background sea clutter without relying on their cooperation [12,13,14].

Recent studies highlighted the enormous potential that the cooperative AIS offers in providing detailed vessel movements and classifying fishing activities [15,16,17], while others investigated its reliability by applying statistical and machine-learning solutions to detect anomaly in maritime behavior [18,19]. AIS position reports can indeed drop out for many reasons, such as weak signals, signal interference in crowded areas or intentionally tampering when entering port or in close proximity of fishery managed areas [20,21]. Moreover, AIS is adopted by only a fraction of vessels (≥15 m in Length Over All).

Regarding non-cooperative systems and maritime traffic surveillance, the satellite Synthetic Aperture Radar (SAR) was considered the more suited if compared with optical remote sensing, as it allows ship detection over wide swaths without being critically affected by weather conditions and day-night cycles [22,23,24,25]. Nonetheless, it lacks regular, global coverage of the oceans [26].

Given their distinct limitations, the synergic exploitation of both the above-mentioned data represents a breakthrough to strongly improve MDA and effectively monitor fishing activities [27,28]. It results in the quantification and additional mapping of the non-reporting ship traffic and gives a more complete and informative picture of vessels’ activity, including Illegal, Unreported and Unregulated (IUU) fishing [29]. The combined use of cooperative and non-cooperative sources has already been proposed in the literature to increase the potential of the single data source in classifying ships [30,31,32], investigating related behavior [33,34] and detecting abnormal activities [35]. Zhao et al. [36] proposed a point-to point association with merge AIS and SAR data using an iterative process based on the minimum distance criteria between the AIS and SAR positions [37], while Mazzarella et al. [33] increased the quality of SAR/AIS fusion by exploiting knowledge of historical vessel positioning information. Park et al. [38] combined for the first time four satellite technologies to publicly reveal large-scale illegal activities by dark fleets operating in North Korean waters. Their method worked only when there were AIS positions relatively close in time to the images, and for many scenes, because of poor AIS reception or vessels not broadcasting, such matching was not possible.

In this context, this study proposes a method to integrate AIS and SAR data, starting from the general approach that was presented by Galdelli et al. [39] and using two types of associations to also address the presence of AIS blackouts. Open-source Sentinel-1 data and the Search for Unidentified Maritime Objects (SUMO) algorithm [40] were used to detect ships and feed an algorithm that was developed to automatically fuse ship positions and fill data gaps due to non-reporting ships that could be hampered by technical limitations or deliberately switching off the system while concealing suspicious activities. SUMO, which works with a faster version of a pixel-based Constant False Alarm Rate (CFAR, [41,42]), was adopted as it represents a good compromise between performance and computational time. A case study in the central Adriatic Sea is analyzed and focus is placed in close proximity of managed areas, such as those surrounding the offshore gas platforms and the 3 nautical miles of the shoreline. Although some aspects of this work are particular to the case study (e.g., presence of offshore platforms and related safety zones), the proposed approach is easily transferable to match AIS and vessel detections from other available global satellite imagery or radar and investigate other managed areas by filling information gaps and revealing activities of “dark” fleets. Achieving a more comprehensive picture of fishing activities at sea is an important step toward sustainable and cooperative fisheries management.

## 2. Materials and Method

**Figure 1:**
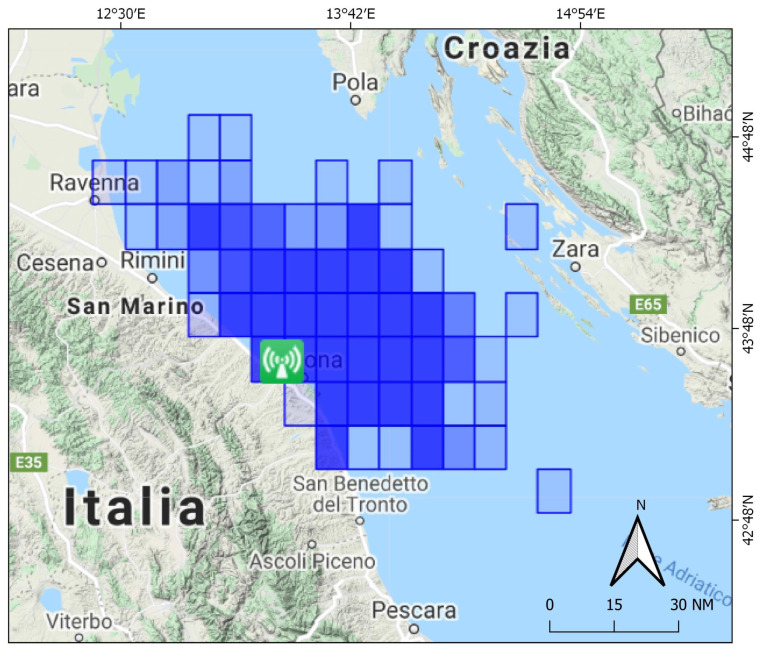
t-AIS data coverage (Source: Marin Traffic, Station #82). The blue-colored areas reflect the quality of data coverage in each grid cell.

Terrestrial AIS (t-AIS) data were collected with a poll rate of 2–5 min from a land-based receiver (Comar SLR300N–PHP dispatcher (http://www.ais.dii.univpm.it, accessed on 12 April 2021)) that is installed on the roof of the Università Politecnica delle Marche, at a height of 205 m above the sea level. Accordingly, the study area was chosen to cover the coverage area (see Figure 1) of selected receiver (a radius of approximately 45 nm around the port of Ancona).

**Figure 2:**
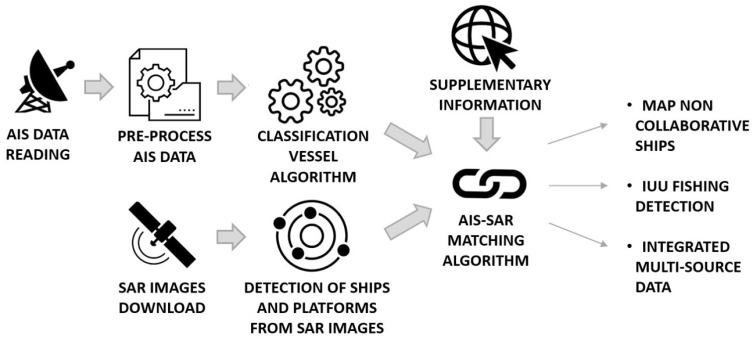
Flowchart to integrate AIS data and SAR images.

This stretch of Adriatic Sea is intensively fished, and the fleet operating in this area includes all fleet segments, from small-scale fishery vessels to large trawlers. Most fishing activity is carried out by bottom otter and rapid trawlers, while the use of set gears (e.g., gillnets, trammel nets, and traps) remains typically confined within 3 nm of the coast (where towed gears have been permanently banned, as defined by Article 13 of EU Council Regulation 1967/2006) and/or in areas unsuitable for trawling [43]. The study area is also characterized by the presence of a few gas platforms, within whose 500 m radius zones it is forbidden to anchor, fish or navigate. The Italian law prescribes the same for all the over one hundred platforms scattered over the Adriatic Sea.

t-AIS data were first pre-processed to generate fishing trips, blackout tracks and investigate their overlay with known managed areas, such as the 3 nm of the coast and the 500 m safety zones in the vicinity of platforms. According to the management measure under investigation and the gear type that is likely to be illegally used, only some AIS report positions were retained and a distance criterion was applied to match them with ship positions from SAR images. To this end, a classification process was carried out to pre-assign AIS transmissions to specific fishing gears (Figure 2).

### 2.1. AIS Data and Processing

#### 2.1.1. AIS Data Pre-Processing

Repeated points and outliers were first removed, as well as pings located in land. t-AIS data were then imported in a PostgreSQL database (https://www.postgresql.org/, accessed on 12 April 2021) by converting geographic coordinates in PostGIS geometries and enriched with a binary in-port status. This was directly derived by the spatial overlay with a 1-km buffer layer and using the *ST_Within* (https://postgis.net/docs/ST_Within.html, accessed on 12 April 2021) function of PostGIS.

Assuming that vessels travel in a straight line, tracks were generated by linear interpolation of subsequent pings and considered to be blackouts if exceeding a predefined threshold of 30 min. Blackouts lasting from 30 min to 2 h were stored in the same database, along with the following metrics inherited/derived from the two vertices (pings) forming the track: date, start and end time, duration, length, positions immediately before and after (vertices of the blackout), speed (as the average of the speeds of the two vertices) and geometry (by default, a line is used). Tracks longer than 2 h were not saved because vessels are unlikely to keep a straight course for long periods [44].

#### 2.1.2. Vessel Classification

Individual trips were identified for each vessel, from the time it leaves a port to the time it returns to a port, and stored in the spatial database. It is worthy to note that often the algorithm failed because of the limited coverage of the data used in this work. Nevertheless, it allowed us to work with single trips and to better identify the type of vessels according to predefined classes: bottom otter trawlers (OTB), beam trawlers (TBB), pelagic trawlers (PTM), purse seiners (PS), longliners (LL) and “OTHER” (including cargo and cruise vessels).

**Figure 3:**
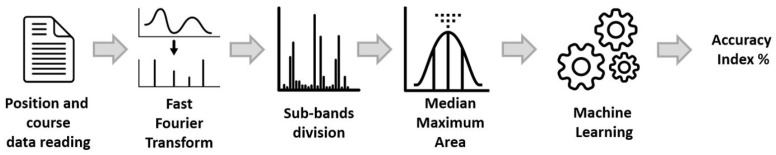
ML approach to classify vessel’s trips (Source: [39]). Reprinted with permission from [39]. 2020 IMEKO.

In particular, a Boosting algorithm (AdaBoostM2, [45]) was executed to classify each individual trip into one of five gear types (Figure 3). The AdaBoostM2 was chosen after performing comparative tests with other ML algorithms and striking the right balance between accuracy and processing time.

Prior to this, a Fast Fourier Transformation (FFT) was applied on position and course data (using the *pspectrum* [46] Matlab function), and the performance was improved by subdividing each spectrum twice into 20 and 100 power sub-bands from which additional features (median, maximum and area of single sub-bands) were extracted and added to the feature space. This choice was given by the right tradeoff between speed of execution and performance obtained.

To validate the vessel classification algorithms, a ground truthed dataset was obtained by experts that manually labeled—with also the support of fleet registers—AIS-based fishing trips that were exerted during 2017–2020.

The machine-learning approach extends what was developed for towed gears in Galdelli et al. [15], with the aim to include non-fishing vessels (as “other”) and additional gear classes such as longlines and purse seines. This was needed to link back the type of vessel, after having matched the SAR target to a specific AIS-based geometry (ping or blackout).

### 2.2. SAR Images and Processing

#### 2.2.1. SAR Images Download

Possible timestamps were identified in the frame 2017–2020, by temporal querying the AIS database and looking for the highest traffic densities. SAR images were then downloaded from two open-source providers—ONDA (https://catalogue.onda-dias.eu/catalogue/, accessed on 12 April 2021) and Copernicus (https://scihub.copernicus.eu/, accessed on 12 April 2021)—according to those identified timestamps, and then further sub-sampled in terms of the number of targets SUMO was able to detect.

It resulted in nine dual-pol (VV + VH) Sentinel-1 acquisitions covering all the study area, although with slightly different footprints. It is due to the orbital motion of the Sentinel-1 satellites.

The image mode was IW GRDH (Interferometric Wide, Ground Range Detected High Resolution [47]), which implies a resolution of 20 m × 22 m (ground range × azimuth) at an Equivalent Number of Looks (ENL) of 4.4.

#### 2.2.2. Detection of Ships and Platforms

**Figure 4:**
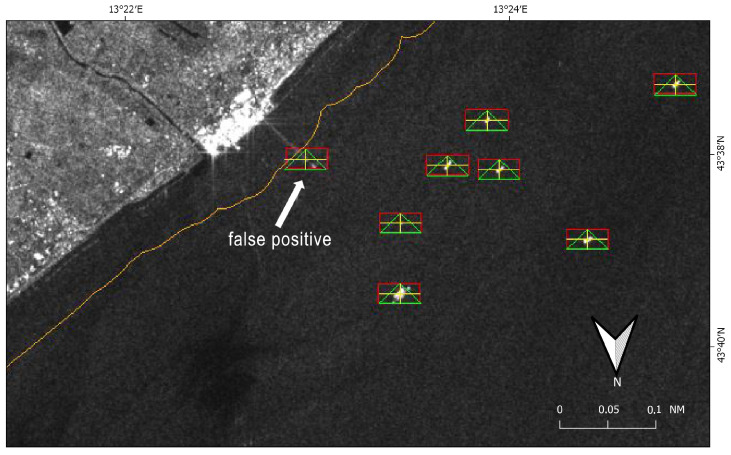
Target detection by SUMO on Sentinel-1 image (9 March 2020 at 05:11:14 UTC), VH channel. The orange line represents the 250 m buffer used by SUMO for land masking, while yellow crosses identify the centroids of the targets which result by merging detections in VH and VV bands (green triangles and red rectangles, respectively). In detail a false positive is shown due to a pier that was not masked by the 250 m buffer.

SUMO was run in fully automatic mode to perform target detection, by working with both the polarimetric bands that constitute the Sentinel-1 satellite image and adjusting different thresholds to deal with the typically irregular distribution of the radar backscatter over a target: 2.3 for VV (vertical transmission and reception) and 1.3 for VH (vertical transmission and horizontal reception). Once identified in each band, neighboring detected pixels were clustered again and thus merged into a single detection result/target (Figure 4).

To avoid inland targets, land masking was applied by using a 250 m buffer of the publicly available Global Self-consistent, Hierarchical, High-resolution Geography (GSHHG) database [48]. The use of this increased land mask buffer size allowed to speed up the image process by overlaying a simpler geometry, and to reduce (but not totally suppress) false alarms due to parts of land that were not accurately covered by the original coastline vector (Figure 4).

Used automatically, SUMO was fast enough to keep up with current high production volumes of Sentinel-1, although it depended for the most part on cross-pol data and thus producing too many false alarms (e.g., signatures of the ship’s wake) that had to be checked. Except for the increased land mask buffer size and the detection threshold adjustments, all the other parameters were set by default.

For each image, SUMO provided as output the list of the georeferenced centroids of each target detected. Given the sensitivity of the VV polarimetric band, only targets that were detected in the VH band were manually retained and passed to the matching algorithm for comparison with the reporting ships. It allowed to further reduce the number of false positives, while minimizing the number of false negative.

### 2.3. Supplementary Information

Positions of the offshore gas platforms were downloaded from the EMODnet–Human Activities web portal [49] and used to confirm those targets that were fixed in repeat-pass imagery as false alarms. They were excluded before passing the detected SAR targets to the matching algorithm. Additional buffer layers were created and used to investigate suspicious behavior, such as those within the 3 nm offshore the coastline and within the 500 m safety zones surrounding the offshore platforms.

### 2.4. AIS-SAR Matching Algorithm

SAR targets were first retrieved from the database, before being filtered to remove overlapping targets and false positives that were likely to be offshore platforms. They were then passed in loop to the matching algorithm. In particular, SAR targets were filtered in two steps by: (i) flagging inter-target distances shorter the 150 m that were manually resolved by retaining only one of the 2 overlapping targets; (ii) excluding SAR targets that fell within 10 m buffers around offshore platforms. This was needed because, in addition to platforms, SUMO generated several false alarms for many reasons, ranging from radio interference and local ocean/atmosphere effects to ambiguities in range/azimuth and situations of very high wind/waves or extremely low backscatter [40].

**Figure 5:**
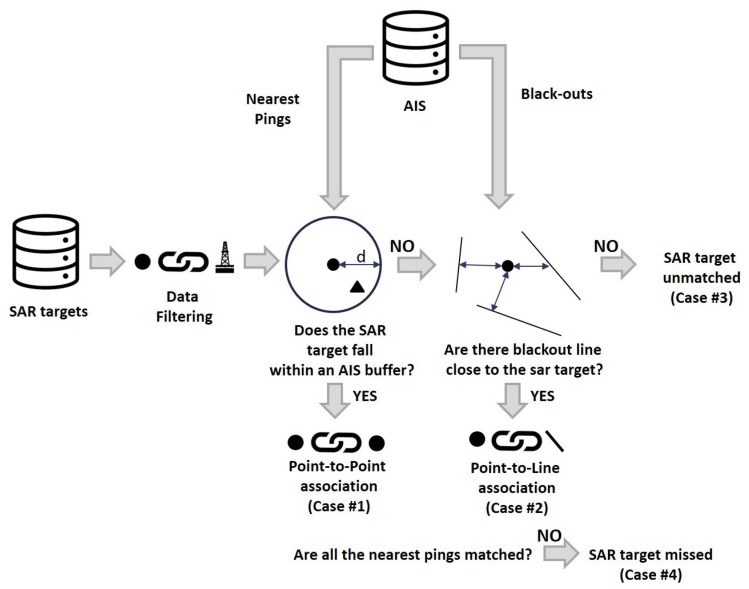
AIS-SAR Matching Algorithm.

As regards AIS data, for each iteration of the loop, pings and blackouts were retrieved at the epoch of SAR image collection: for each Maritime Mobile Service Identity (MMSI), only nearest (in time) and preceding pings were retained (as compared to the SAR image epoch), while for blackouts start and end times were used to select only lines straddling the SAR image epoch. Selected AIS data were then used to attempt a Point-to-Point or Point-to-Line associations, according to the following main cases under which the SAR target can fall (Figure 5):

- **Case #1 Point-to-point**: the SAR target is within a buffer area centered on AIS data. Retrieving all the AIS pings temporally closest to the SAR image, buffer sizes are set proportional to the distance *d* each vessel can travel in the time interval ΔT from the AIS transmission to the image acquisition. Taking into account that vessels can increase their speed during ΔT, the buffer radius is increased by 20%:
  (1) d=(vΔT)∗1.2
  where *v* is the speed the vessel broadcasted via AIS.
  In case of SAR targets falling within multiple buffers, the choice was made based on the hypothetical positions that vessel could reach at SAR epoch and the target was associated with the nearest buffer edge. This occurs in areas of high traffic density where vessels are close to each other.
- **Case #2 Point-to-line**: A SAR target is detected but no AIS reports are available in the vicinity. This could be caused by a transmission problem or a voluntary switching off.
  An attempt was done to associate the SAR target to the nearest blackout, by creating a variable size buffer whose radius *r* increases moving away from the SAR epoch.
  The buffer was generated dynamically by moving a pointer along the blackout line (ρ*_i_*) according to *N* regular intervals *d_i_* that were defined as:
  (2) $$d_{i}=\Delta v*\Delta T_{i}$$
  (3) $$N=\frac{duration\;of\;blackout}{AIS\;poll\;rate}$$
  where Δv is the average speed between the two AIS vertices of the blackout and *ΔT_i_* is the time-lag between *T_i_* and the SAR epoch.
  (4) $$T_{i}=T_{start\_blackout}+\left(i*AIS\;poll\;rate\right)$$
  *i* starts at zero (starting vertex) and increase to N (ending vertex), while *d_i_* ranges between zero (when *T_i_* = SAR epoch) and maximum values that are reached at the vertices. The value of the buffer radius is determined accordingly, and defined as:
  (5) $$r_{i}=\frac{l}{4}e^{-\frac{{\left(d_{i}-\mu \right)}^{2}}{2\sigma^{2}}}$$
  where μ *e* σ are the mean and standard deviation of the *N* hypothetical distances *d_i_* travelled by the vessel (and the pointer) while moving between the 2 vertices of the blackout line.
  Again, in case of SAR targets falling within multiple buffers, the closest buffer is selected for the Point-to-Line association.
- **Case #3 No match**: The SAR target is unmatched because the AIS data is not available. It is due to a remaining false alarm, a blackout longer than 2 h or a vessel that is not adopting AIS.
- **Case #4 No match**: A SAR target is missed even if AIS data is available, due to the failure of the vessel detection. This may occur for several reasons, including small boats that were not detected by SUMO or bugs/shortfalls of the algorithm itself.

For each target, both Cases #1 and #2 were checked before labeling the SAR detection as unmatched (Cases #3). At the end of the *for* loop, all the non-associated AIS data were assigned to Cases #4.

Both Cases #1 and #2 validate the performance of the SAR-based detection algorithm, whereas Case #2 discriminates collaborative and non-collaborative ships, playing a key role in detecting potential suspect behaviors.

The pseudo-code that implements the AIS-SAR matching algorithm is summarized in Algorithm 1 as it follows:
**Algorithm 1:** AIS-SAR Matching Algorithm.
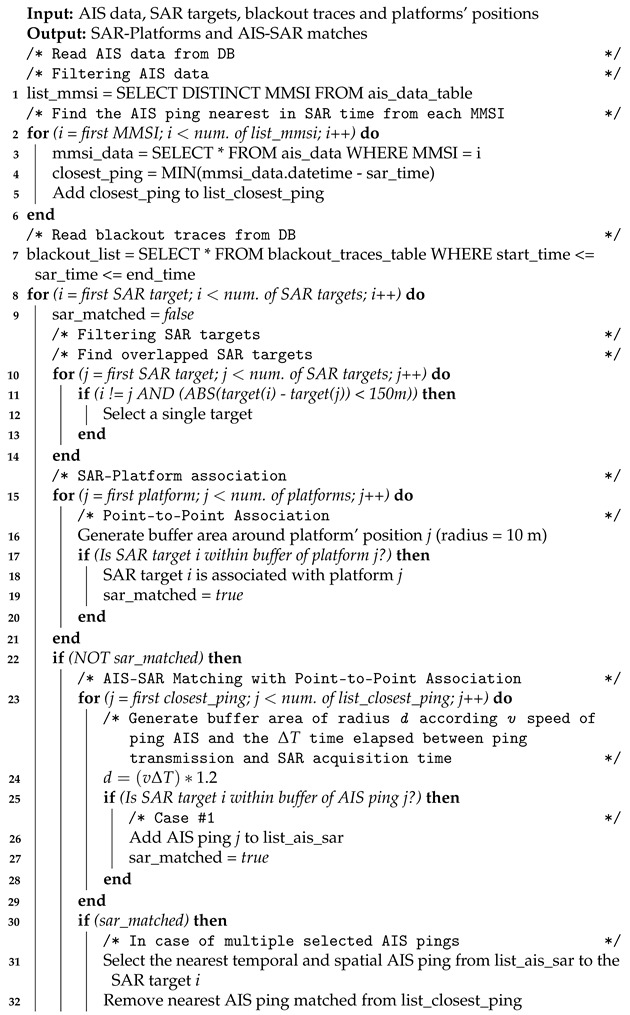

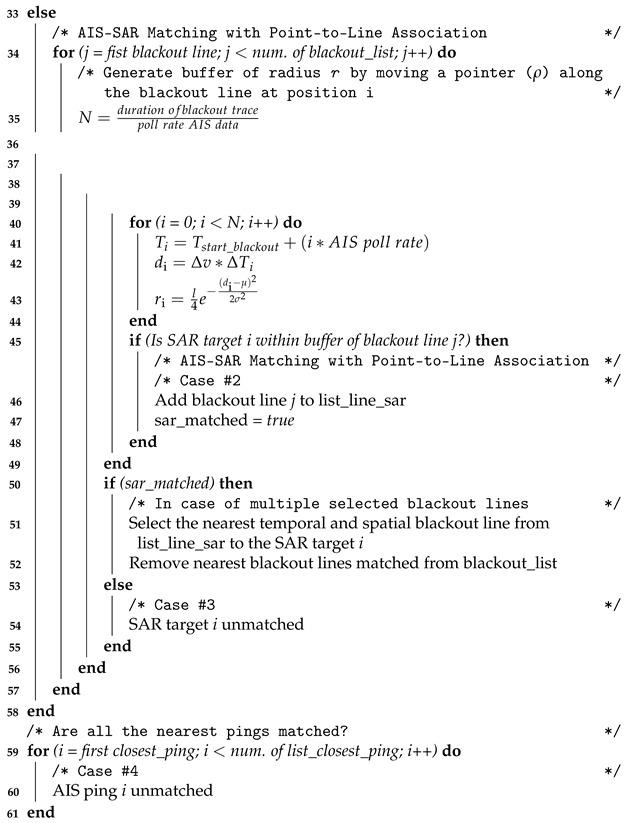


## 3. Results

Nine dual-pol (VV + VH) Sentinel-1 acquisitions over the study area were processed and results are shown in Table 1. Tests were performed using Python on board a PC i7-7700HQ, 16 GB of RAM and NVDIA GeForce GTX 1050. It took 5 min to process each Sentinel-1 image, while the AIS-SAR matching algorithm required ∼40 s to process each target list and return AIS-SAR correlations.

For each processed image, selected AIS blackouts and nearest pings are reported, excluding those broadcasted by vessels in maneuver or moored in ports. Several reporting ships were indeed docked at the various ports in the images, but those ships were not detected by SUMO since the ports fell inside the land mask. These reporting ships were therefore excluded from the evaluation.

Given the limited coverage of the t-AIS data, only targets identified by SUMO within the 45 nautical miles (receiver reception distance) were retained. Out of these, large numbers of detections were deemed azimuth ambiguities by SUMO and automatically assigned the lowest reliability level and dropped. Such numbers are typical for coastal areas that often show many azimuth ambiguities from port and urban constructions. Additional false positives were filtered out by the algorithm because overlapping and/or associated with platforms (FP and SP columns, respectively, Table 1), and therefore not considered for comparison with the reporting ships. Finally, the fusion process between the SUMO detected ships and the selected AIS data returned point-to-point and point-to-line correlations (PP and PL columns, respectively, Table 1). Out of the associated blackouts, a few lines were considered suspicious and worthy of further investigation (SB column, Table 1).

**Figure 6:**
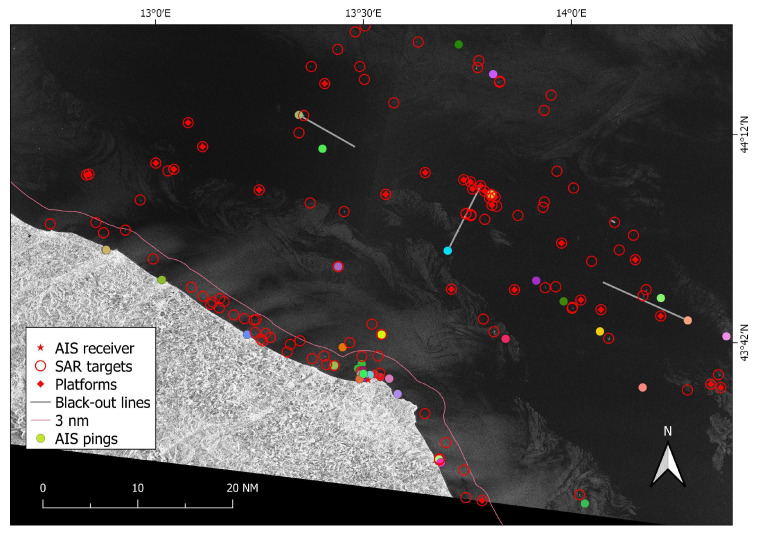
VV polarization of the Sentinel-1 image used as example, SAR targets (red circles) and selected AIS data (colored points and grey lines). AIS positions are categorized by MMSI.

The rest of this section presents an end-to-end example of the proposed analysis. The Sentinel-1 image under study was taken on 19 July 2019 at 05:10:31 UTC (Figure 6) over the central coast of the Marche Region (Italy).

Once gears were assigned, all AIS report positions were retained (regardless of the type of gear in use) and consisted of 46 nearest AIS pings transmitted by different vessels (unique MMSI) classified as: 18 OTB, 15 PTM, 3 TBB and 10 “other” (cruise/cargo). Ground truth revealed that these AIS transmissions belonged to 46 fishing trips that were labeled by experts as 16 OTB, 15 PTM, 5 TBB and 10 “other”, reflecting the mean accuracy of our classification that reached 90%, 85.7%, 90% and 85.7%, for the over mentioned gear classes, respectively.

**Figure 7:**
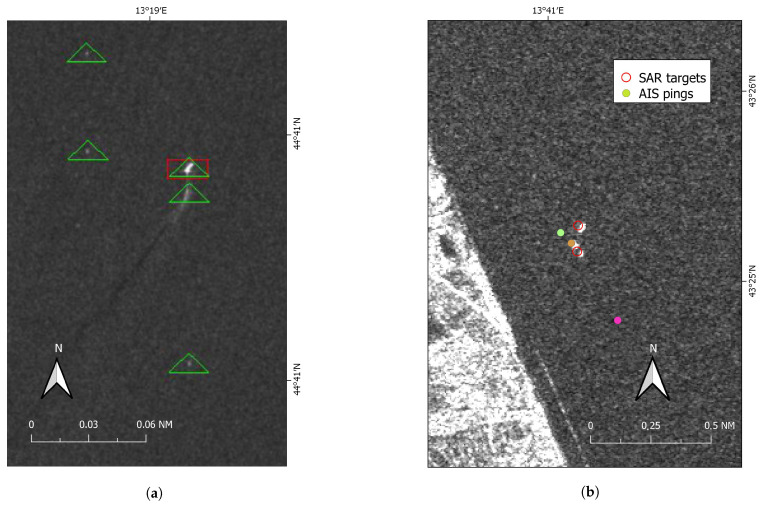
Sentinel-1 image taken on 2019-07-19 and a false positive due to a ship’s wake (inter-target distance ≤150 m). Green triangle and the red rectangles are objects detected in VH and VV, respectively (**a**). Close-up view of the 3 nm costal area (VH channel). 3 AIS positions were reported by a PTM (green ping), a cargo/cruise (orange ping) and a small OTB (pink) that according to its AIS, was below the limit of detectability for SUMO (**b**).

Twenty reporting ships were maneuvering or anchored in port and therefore excluded from the following AIS-SAR integration. The automatic SUMO analysis gave 253 detections in the image. Out of these, one false positive was due to a cross-pol signature of a ship’s wake (Figure 7a), 117 were within the radius of 45 nm around the Comar receiver, and 28 were linked to offshore platforms.

The matching algorithm between the remaining 116 SUMO detected ships and the 26 AIS positions returned 21 point-to-point associations (Case #1) with pings broadcasted by 8 OTB, 7 PTM, 1 TBB and 5 “other” vessels. Additionally, 5 reported AIS positions were left uncorrelated as no SAR targets were in their immediate area (Case #4). Regarding these pings not correlated with SAR detections, three of them were small boats according to their AIS (13, 14 and 18 m in length), which are apparently below the limit of detectability for SUMO (at the adopted Nominal false alarm rate P_FA_, Figure 7b).

**Figure 9:**
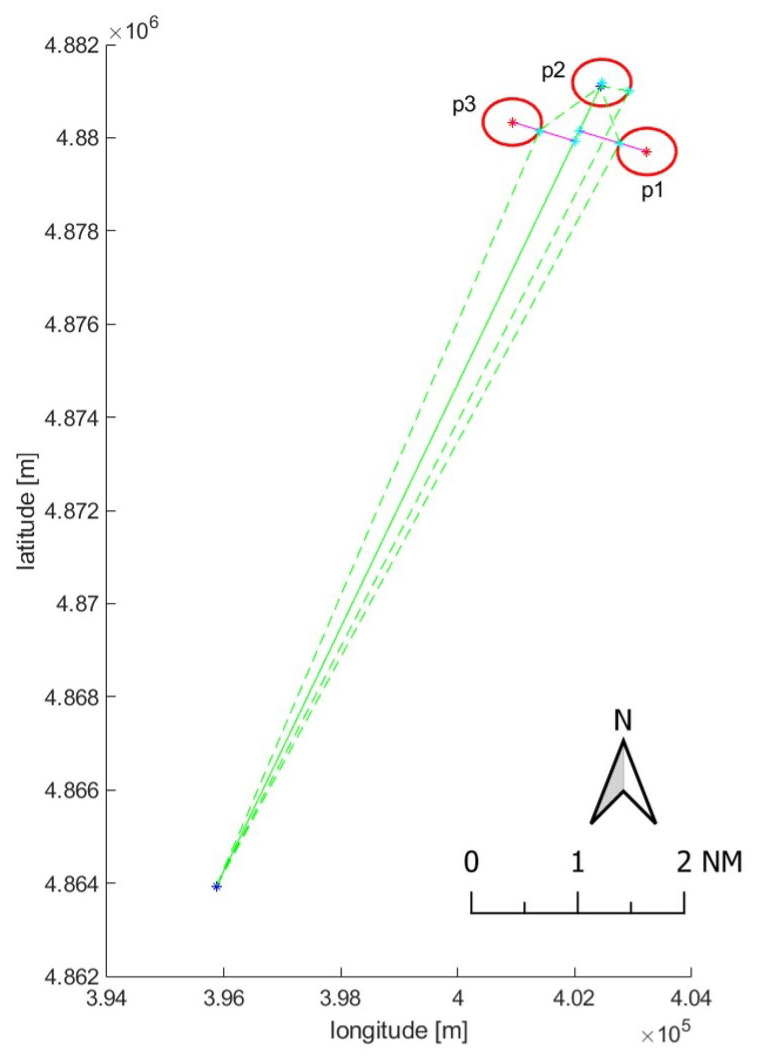
Graphical representation of a suspicious blackout (green line) in proximity of an offshore gas field (platforms p1, p2 and p3). Blu points consist of the latest ping before the switching off and first available ping after the power on, while cyan markers are the contact points between the 500 m safety zones (red circles) and the minimal potential routes (dashed green lines) that the vessel could follow to enter the forbidden areas.

All the 8 blackouts resulted in point-to-line associations. Out of these, one (42 min in duration and ∼18 km in length) belongs to a vessel that behaved suspiciously entering the safety zone surrounding the offshore platform p2 and navigating very close to other two platforms (1.2 km from platform p1 and 1.1 km from platform p3, Figure 8 and Figure 9).

**Figure 10:**
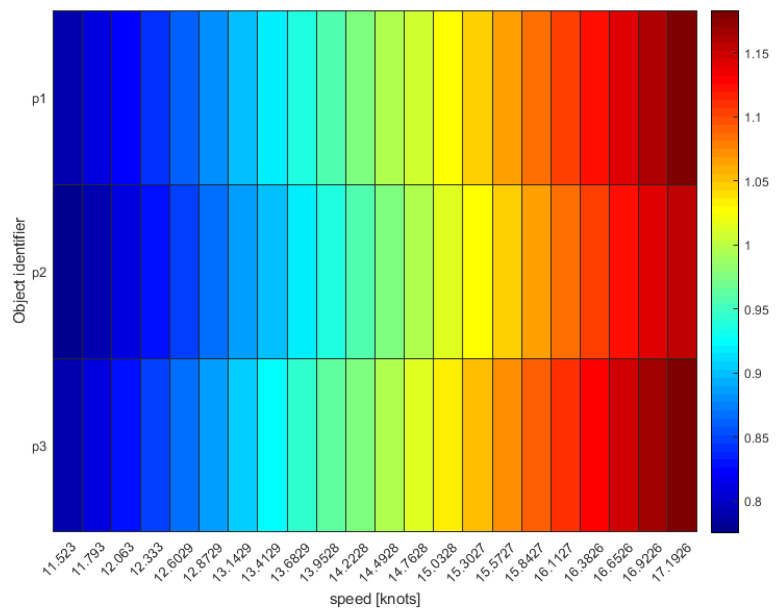
Graphical representation of the estimated distance travelled by vessel towards the offshore platforms p1, p2 and p3.

**Figure 11:**
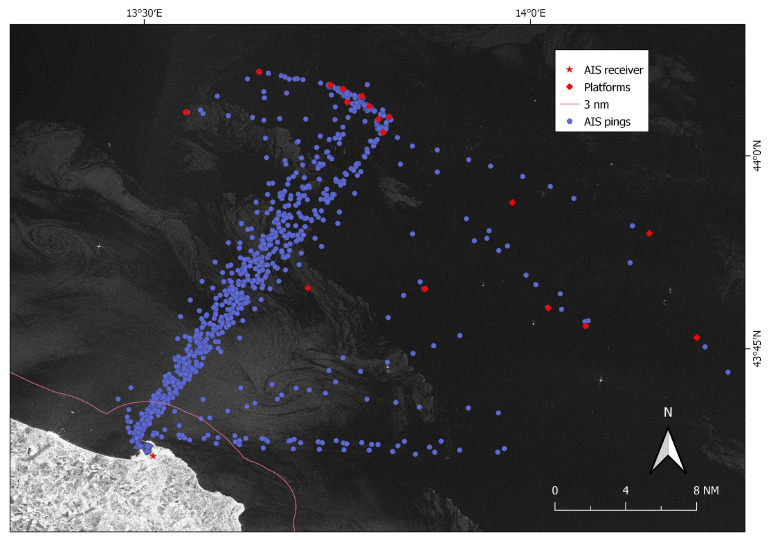
Sentinel-1 image taken on 19 July 2019 (VV channel) and AIS signals (purple points) broadcasted by a platform supply vessel during July 2019.

The heat map in Figure 10 helped investigate the behavior of this vessel and its potential speed values during the blackout, with rows inform on the ratio between the potential travelled distance at different speeds and the minimum distance to reach/exit each forbidden area (pink lines in Figure 9) of platforms p1, p2 and p3. In line with the high/non-fishing speed values predicted by the heat map, the classification algorithm labeled this vessel trip as “other”, and the plot of its monthly activities confirmed it was a tugboat acting as platform supply vessel (Figure 11).

## 4. Discussion and Conclusions

The growing number of ship-reporting technologies and remote-sensing systems are generating an overwhelming amount of spatio-temporal and geographically distributed data related to vessels and their movements. The integration of these data is of paramount importance to fill data gaps and have a better picture of what is happening at sea.

AIS may accidentally malfunction or be voluntary switched off. Nevertheless, it can highlight non-reporting vessels and be helpful to detect suspicious fishing activities especially when coupled with satellite imagery.

In this context, a workflow is presented to integrate AIS and SAR data. For each Sentinel-1 image, the proposed algorithm ingested the list of detections, retrieved AIS positions and blackouts and attempted two type of correlations (Cases #1 and #2), before labeling remaining SAR targets and loaded AIS data as unmatched (Cases #3 and #4, respectively). The approach was tested with a series of Sentinel-1 images, reporting resolved point-to-point and point-to-line correlations (PP and PL in Table 1) that validate detections in satellite imagery, and seeking ships that were-intentionally or not-not reporting their positions (SB in Table 1). Obviously before data ingestion, AIS positions and SAR targets were filtered to take account of the limited antenna coverage and “in port” AIS transmissions. Suspicious point-line associations were finally investigated, especially when they occurred in proximity of banned or fishery-regulated areas, such as those surrounding the offshore gas platforms and the 3 nautical miles of the shoreline. To this end, a machine-learning classification was needed and carried out to pre-assign AIS transmissions to specific classes and fishing gears. Other research experiences have proposed similar AIS-SAR data associations [31,33,50], but none of them attempted to correlate blackouts by point-to-line associations. It allowed to advance suspicions on the behavior of ships by retrieving information from areas where AIS blackout periods occur.

More specifically, results were better shown for one of the processed satellite SAR images (19 July 2019 at 05:10:31 UTC). For this Sentinel-1 image, the algorithm performed well in associating all the known 28 offshore platform positions with the detected targets. Out of the remaining 89 targets detected by SUMO, one was on a bright ship wake (occurring in addition to the detection of the ship itself) and it was removed before feeding the algorithm, and 59 fell into Case #3 and were labeled as unmatched. Visual inspection gave no sound reasons to believe they were not a ship not adopting AIS. Of course, some of them might still be false alarms that could be deleted by repeating target analysis.

Five AIS pings were not correlated with detections (Case #4) since there were no SAR targets that fell in their buffer areas. Visual checking confirmed that they were below the limit of detectability of the Sentinel-1 image for SUMO: even if some of them showed a very weak signature, it was always at the level of the clutter. Twenty-one SAR targets fell in Case #1 and were correctly point-to-point matched with corresponding AIS pings, while for the 8 point-to-line associations (Case #2) only assumptions could be made on the accuracy of the results as AIS was actually off. One of these blackout lines was later labeled as “other” and clearly traced by a platform supply vessel that was thus non-violating the managed 500 m safety area. This was also confirmed by the analysis of the heat map (Figure 10), which indicated only values higher than 11 knots as possible speeds to cover the blackout distance in the considered time. This speed values are too high to be related to fishing activities, which are performed at 6/8 knots maximum (by “rapido” trawlers). Nevertheless, the proposed hypothetical route analysis proved useful to investigate unreported positions/blackouts that could hinder suspicious behaviors in proximity of regulated areas.

Except for the machine-learning classification that was validated against fleet registers and expert knowledge, the quality of the AIS-SAR matching algorithm was very difficult to evaluate because no ground truth was available. It was not easy either to create and to be sure about it.

Moreover, in the first place, accuracy relies on the performance of the SUMO detector and of the SAR imaging itself, which depends in turn on many variables that are related to the targets (e.g., ship size, ship material), the sensor (e.g., resolution, polarization), the environment (e.g., wind, waves) and the imaging geometry (e.g., incidence angle, aspect angles). Nonetheless, visual inspections validated associations and suggested that the proposed approach could help monitor fishing activity and rate the effectiveness of some fishery-regulated areas, provided that SAR imagery is available. Satellite images are indeed limited to a maximum of a few images per day and cannot capture the same area every day due to satellite orbital cycles. It hampers the monitoring of the maritime situation on a continuous basis.

Future work is planned to try to fix issues that emerged in this initial development, starting from the detection of the coastline that will be improved by applying a state-of-the-art wavelet-based methodology [51]. This will allow the minimization of the number of coastal infrastructures that fell outside the land mask and were detected as false alarms. Moreover, different data formats will be tested as input and processed in SUMO, with special concern on TerraSAR-X and Cosmo-Skymed whose improved resolutions and polarimetric capabilities could improve ship detection and consequently result in a higher number of associations. Furthermore, their different/non-synchronized revisit times will allow frequent enough matching to really monitor fishing fleets. Combing different satellite technologies with different revisit times and spatial coverages, these investigations could indeed be performed at large enough spatial and temporal scales to enable transparent fisheries monitoring.

## Figures and Tables

**Figure 8:**
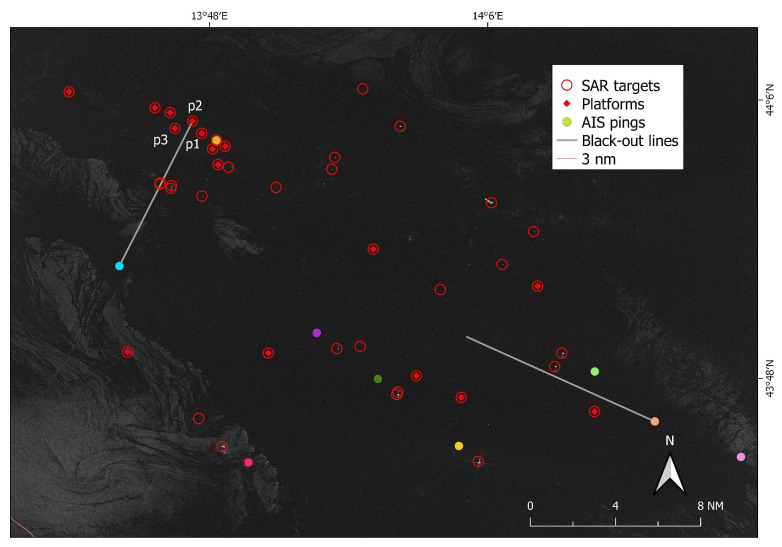
Close-up view of the Sentinel-1 image taken as example (VV channel), overlaying 41 SUMO/SAR detections, 9 AIS reported positions, 3 AIS blackouts and 6 offshore platforms. The starting vertex of the blackout entering the managed area of platform p2 is at the same time (for the given MMSI) the ping nearest to the SAR epoch (cyan AIS position).

**Table 1:** Summary of target detection and correlation results for all the processed images.

Datetime	AIS (in Port *)	SUMO (45 nm **)	BL ^1^	FP ^2^	SP ^3^	PP ^4^	PL ^5^	SB ^6^
20 March 2017 16:56:52	85 (44)	290 (102)	2	1	18	28	2	1
19 July 2019 05:10:31	46 (20)	253 (117)	8	1	28	21	8	3
20 July 2019 16:57:09	48 (28)	128 (37)	8	0	14	7	8	1
24 July 2019 05:18:43	83 (52)	222 (57)	11	2	10	18	11	0
25 July 2019 05:11:14	88 (65)	195 (70)	14	2	28	20	14	0
26 July 2019 16:57:52	47 (21)	144 (39)	3	1	17	11	3	0
9 March 2018 16:57:40	49 (23)	147 (39)	3	0	17	8	3	0
9 March 2019 05:10:32	48 (20)	228 (116)	1	2	36	20	1	0
9 March 2020 05:11:14	79 (48)	321 (187)	5	2	28	28	5	0

* AIS pings in port (transmitted by maneuvering or moored vessels). ** SAR Targets within 45 nm. 1 BL = Blackout lines. 2 FP = False positive. 3 SP = SAR-Platform associations. 4 PP = Point-to-Point associations. 5 PL = Point-to-Line associations. 6 SB = Suspicious blackouts, within 500 m safety areas or 3 nm.

## Data Availability

Publicly available satellite images were analyzed in this study. This data can be found here: ONDA: https://catalogue.onda-dias.eu/catalogue/ (accessed on 12 April 2021) and Copernicus: https://scihub.copernicus.eu/ (accessed on 12 April 2021). Restrictions apply to the availability of AIS data. Data was obtained from a custom AIS station that is interfaced with Marine Traffic.
